# Possible effect of mutations on serological detection of *Borrelia burgdorferi* sensu stricto ospC major groups: An in-silico study

**DOI:** 10.1371/journal.pone.0292741

**Published:** 2023-10-10

**Authors:** Samir Mechai, Heather Coatsworth, Nicholas H. Ogden

**Affiliations:** 1 Public Health Risk Sciences, National Microbiology Laboratory Branch, Public Health Agency of Canada, St Hyacinthe, QC, Canada; 2 Groupe de Recherche en Épidémiologie des Zoonoses et Santé Publique (GREZOSP), Faculté de Médecine Vétérinaire, St Hyacinthe, QC, Canada; 3 One Health Division, National Microbiology Laboratory Branch, Public Health Agency of Canada, Winnipeg, MB, Canada; University of Kentucky College of Medicine, UNITED STATES

## Abstract

The outer surface protein C (OspC) of the agent of Lyme disease, *Borrelia burgdorferi* sensu stricto, is a major lipoprotein surface-expressed during early-phase human infections. Antibodies to OspC are used in serological diagnoses. This study explored the hypothesis that serological test sensitivity decreases as genetic similarity of ospC major groups (MGs) of infecting strains, and ospC A (the MG in the strain B31 used to prepare antigen for serodiagnosis assays) decreases. We used a previously published microarray dataset to compare serological reactivity to ospC A (measured as pixel intensity) versus reactivity to 22 other ospC MGs, within a population of 55 patients diagnosed by two-tier serological testing using *B*. *burgdorferi* s.s. strain B31 as antigen, in which the ospC MG is OspC A. The difference in reactivity of sera to ospC A and reactivity to each of the other 22 ospC MGs (termed ‘reactivity difference’) was the outcome variable in regression analysis in which genetic distance of the ospC MGs from ospC A was the explanatory variable. Genetic distance was computed for the whole ospC sequence, and 9 subsections, from Neighbour Joining phylogenetic trees of the 23 ospC MGs. Regression analysis was conducted using genetic distance for the full ospC sequence, and the subsections individually. There was a significant association between the reactivity difference and genetic distance of ospC MGs from ospC A: increased genetic distance reduced reactivity to OspC A. No single ospC subsection sequence fully explained the relationship between genetic distance and reactivity difference. An analysis of single nucleotide polymorphisms supported a biological explanation via specific amino acid modifications likely to change protein binding affinity. This adds support to the hypothesis that genetic diversity of *B*. *burgdorferi* s.s. (here specifically OspC) may impact serological diagnostic test performance. Further prospective studies are necessary to explore the clinical implications of these findings.

## Introduction

Until recently, a two-tier testing approach, combining an initial enzyme-immuno assay (EIA) followed by a Western immunoblot (WB) has been recommended for serological diagnosis of Lyme disease in North America [1]. If the first test is negative then no further investigations are recommended, but if the EIA is positive then the WB is performed to confirm the results (https://www.cdc.gov/lyme). Results of a WB for IgM antibodies are used for diagnosis in early infection and results of a WB for IgG antibodies are used during the later stages of the disease [2]. For both the IgM and IgG immunoblots, responses to the 21 kDa outer surface protein C (OspC) contribute to a positive test result [1, 3]. However, serological diagnosis of Lyme disease is not simple, especially during the early localized stage of the infection which occurs within 1–28 days after the tick bite [4]. With low levels of antibodies in early infection (when treatment may be simplest and most effective; [5]) 60% to 70% of infections at this stage are undetectable by serological diagnosis [6]. A number of suggestions for improving serodiagnosis of Lyme disease include the addition of other outer surface proteins to the WB algorithm [7], and eliminating the subjectivity of WB interpretation by using single serological assays based on proteins such as VlsE and C6 [6, 8]. Most recently, a two-step EIA approach, with similar performance as the EIA-WB two-tier approach, has been approved by the FDA [9] and Health Canada. *Borrelia burgdorferi* sensu stricto is a diverse bacterium [10] and its genome encodes over 1,400 proteins, including those that are immunogenic outer surface proteins used in diagnostic testing. It has been suggested that some proteins used in serological assays, including WBs, are not expressed identically amongst strains, and antibodies raised against one strain may bind with lower affinity to the proteins of another strain used as antigen in the assay, increasing the likelihood of false negative results [11]. If so, then using a combination of strains to provide antigens may reduce the risk of false-negative test results.

OspC is a protein essential for the early stages of *B*. *burgdorferi* infection of mammals [12], and is amongst the first major surface proteins to elicit antibodies during infection [13]. OspC sequences are frequently used for strain typing with, at the time of writing, 47 ospC major groups (MGs) and subtypes known for *B*. *burgdorferi* s.s. in North America [14]. Strain diversity is not fully captured by ospC sequences, and there are 162 sequence types (STs), identified for this species using concatenated housekeeping genes in a multi-locus sequence typing (MLST) scheme (https://pubmlst.org/). Nevertheless, strain typing by ospC MGs has robust associations with typing by MLST and whole genomes, and these taxonomic groupings may be associated with ecological drivers of selection including reservoir host associations [10, 15, 16]. In general, studies suggest that for *B*. *burgdorferi* there is limited cross-immunity following infection with different strains when strain typing is based on ospC MGs [17, 18], and experimental studies identify variable capacity of ospC MGs to induce antibody responses that react with heterologous strains [19, 20].

Here we explore the possible effects of genetic diversity of *B*. *burgdorferi* on serological assays using an in-depth look at the diversity of OspC and, from a previous study, possible impacts on WB signals. In particular we explore if antibodies raised against an infecting strain bind with lower affinity to the OspC A of the B31 strain used as antigen in the WB [21]; the genetic distance between the infecting strain and reference B31 strain could be a useful measure of the likely sensitivity of the WB test for the infecting strain [22].

We explored the genetic distance of the different ospC sequences of 82 whole genomes from North America, from ospC A of the B31 strain. Using the combined ospC sequence data and WB band intensity data in [23] we tested the hypothesis that the sensitivity of the serological test decreases the higher the genetic distance of infecting strains from the B31 strain that provided the antigen in the diagnostic test. We explored genetic distances of the whole ospC sequences, and subsections of the sequences as explanatory variables of differences in reactivity of sera to antigen of different ospC MGs. To support the idea that phylogenetic distance would be associated with variations in the primary structure of the OspC protein, we conducted a single polymorphism analysis of the ospC sequences.

## Material and methods

### Samples and data used

Of the strains used in this study for phylogenetic analysis, 64 were isolated from host-seeking ticks (*Ixodes scapularis*) collected by drag sampling in three Canadian regions: southeastern Manitoba, northwestern Ontario and Nova Scotia [10]. The Sequence Read Archive (SRA) data are available in GenBank (https://www.ncbi.nlm.nih.gov/) through the BioProject accession number: PRJNA416494. Additional strains download from Genbank were PAli (NZ_CP019845.1), PAbe (NZ_CP019917.1), MM1 (NZ_CP031398.1), 297 (NC_018983.1), B31 (NC_001903.1), 118a (NC_012261.1), 156a (NC_011853.1), 29805 (NC_012513.1), 72a (NC_011974.1), 94a (NC_012263.1), Bol26 (NC_012512.1), CA-11-2A (NC_012157.1), JD1 (NC_017395.1), N40 (NC_017401.1), WI91-23 (NC_012151.1), ZS7 (NC_011724.1), B331 (NZ_CP017202.1) and B31_NRZ (NZ_CP019755.1).

In a study by [23] conducted using North American samples, the sera from 55 patients diagnosed with Lyme disease were used to explore antibody cross-reactivity against an array created with OspC proteins expressed in the laboratory. The array comprised one example of each of the 23 ospC MGs: A (X69596.1), A3 (EF592541.1), B (CP001422.1), C (DQ437462.1), C3 (EF592543.1), D (CP001484.1), D3 (EF592544.1), E (AY275221.1), E3 (EF592545.1), F (L42896.1), F3 (EF592547.1), G (AY275223.1), H (CP001271.1), H3 (FJ932733.1), I (AY275219.1), I3 (FJ932734.1), J (CP001535.1), K (AY275214.1), L (EU375832.1), M (CP001550.1), N (EU377775.1), T (AY275222.1) and U (CP001493.1). The GenBank accession number of these data is GSE45996. In the study, sera from the patients probed the proteins of the array and antibody binding was measured using pixel intensity obtained by image analysis of scanned, probed arrays. For each ospC MG in the array, the human sera produced a range of pixel intensity values, and correlation coefficients (r, log10 transformed) for these pixel intensity values (for simplicity referred to as ‘reactivity’ in the following) were obtained for each pair of ospC MG used in the array as an estimate of antibody cross-reactivity between the ospC MGs. Baum and colleagues found that antibody cross-reactivity correlation coefficients were explained only in part by overall amino acid identity of the ospC MGs, more so by amino acid identity of the C-terminus region of the proteins. The ospC MGs in the strains infecting the 55 patients were unknown, but the patients had all been diagnosed with Lyme disease by serodiagnosis using a two-tier EIA and WB for which the antigens were obtained from *B*. *burgdorferi* s.s. strain B31, as described in [24] (an earlier study that used the same patient sera as in [23]), which carries the ospC A MG. These data provide a range of reactivity values to each ospC MG used in the array [23] in an infected population diagnosed by serological diagnosis that used B31 antigen with the ospC A MG. Therefore, the data provide the opportunity to explore if genetic distance between ospC MGs and ospC A could explain differences in the ranges of reactivity of the sera to the different ospC MGs.

### Phylogenetic analysis

To explore the relatedness of ospC sequences that may underly differences in reactivity of sera, a number of phylogenetic analyses were conducted. First, a Bayesian maximum likelihood (ML) phylogenetic tree was built using 64 sequences published in a previous study [10] and 113 other nucleotide sequences downloaded from GenBank, corresponding to 31 ospC MGs and 16 ospC subtypes. The tree was constructed using MrBayes 3.2.6 [25] with Geneious Prime 2019, where ospC X (accession number: HM047876) was used as outgroup to produce a robust tree topology, because this ospC MG is rare and particularly genetically different from the other ospC MGs known in North America [26]. The multiple nucleotide sequence alignment used to build the tree was generated using MAFFT v7.450 [27, 28] under Geneious Prime 2019. This global tree served to illustrate that the 23 ospC MGs used by Baum and colleagues are representative of the known variation in ospC sequences.

Second, the nucleotide sequences of the 23 ospC MGs used by [23] as genomic template for cloning ospC MGs were aligned using MAFFT algorithm v7.450 in Geneious Prime 2019 with a default setting in order to explore the smallest number of evolutionary events occurring within a coding subsection of interest [29]. Third, Neighbor-Joining trees of the ospC sequence alignment and multiple alignments of nucleotide sequences of each of the sequences of the different amino acid (aa) sequence subsections forming the OspC protein were constructed using near-minimal sum of branch-length (i.e., Hamming distance) [30] in PHYLOViZ V2.0 [31]. These sequence subsections and corresponding deduced amino acid sequences are: N-terminus [aa: 19–41], α1 [aa: 42–74], L1 [aa: 75–78], β1 [aa: 79–82], L2 [aa: 83–84], β2 [aa: 85–89], L3 [aa: 90–94], α2 [aa: 95–111], L4 [aa: 112–120], α3 [aa: 121–145], L5 [aa: 146–151], α4 [aa: 152–159], L6 [aa: 160–169], α5 [aa: 170–196], C-terminus [aa: 198–210] [32]. These sequences were assembled into nine sub-sections of the ospC sequence: the five α-helices linked by three loops L4, L5 and L6 (i.e., α1, α2L4, α3L5, α4L6 and α5), two β-strands connected by loops L1, L2, L3 (L1β1L2β2L3), and the N-terminus, pepC10 and C-terminus.

For the 22 ospC MGs in the array of [23], the genetic distance of nucleotide sequences from the ospC A MG (for the whole sequence and subsections described above) was computed using the Substitution and Indel Distances to Infer Evolutionary Relationships (SIDIER) package in R 4.1.3 (https://www.r-project.org/) [33]. Most inference methods use only nucleotide substitutions to infer taxa, whereas the SIDIER approach combines both indel and substitution matrices to compute the genetic distance which is important in case of very closely related and gapped sequences [34]. The multiple nucleotide sequence alignments (MSA) were generated using MAFFT v7.450 [27, 28] under Geneious Prime 2019 with default settings. Alignments were trimmed to the shortest sequence length to avoid sequence end gaps in computing the genetic distance matrices.

### Statistical analysis

The outcome variable was the difference in reactivity to ospC A and reactivity to the other 22 ospC MGs (termed reactivity difference). The null hypothesis is that any variations in reactivity difference would be random, and unrelated to the genetic distance between an ospC MG and ospC A. The alternative hypothesis is that the reactivity difference increases with genetic distance, suggesting that the sensitivity of the serological test decreases/increases (i.e., with pattern) with the genetic distance of infecting strains from the B31 strain. Therefore, if the null hypothesis is rejected, then the associations between genetic distance (i.e., between an ospC MG and ospC A) and reactivity difference would signal a possible impact of genetic diversity on serological test sensitivity, at least in terms of responses to OspC.

To test these hypotheses, regression models were developed in Stata V15.1 [35]. Different regression models were tested to identify if the different structural fragments of the OspC molecule are capable of better explaining observed variations in reactivity difference. The explanatory variables were the genetic distance of the whole ospC sequence from ospC A, as well as similar estimates of genetic distance using the 8 subsections of the ospC sequences described above. The C-terminal conserved region corresponding to the pepC10 subsection used in the new two-EIA diagnostic tests [9] was tested as a covariate separately from the C-terminus variable in different models to avoid collinearity. For statistical analysis using the C-terminus subsection, reactivity difference for only 20 ospC MGs were used because the ospC N (EU377775.1) and L (EU375832.1) sequences do not have this subsection. Exploring impacts of genetic variation by segment allowed identification of the fragments that most explained variation in reactivity difference. The adjusted R squared was used to evaluate how the models changed when changing predictors qualitatively (i.e., type of fragment) and quantitatively (i.e., number of fragments) in the analyses. Standard regression diagnostics were conducted, and multinomial terms were used to explore non-linear relationships between reactivity difference and genetic distance. A Kernel-weighted local polynomial smoothing method was used to fit relationships with higher-order polynomials (i.e., > 3 terms), but for models requiring complex multinomial terms, genetic distance was replaced by deduced phylogenetic groups as a categorical explanatory variable.

### Single-nucleotide polymorphisms detection

The importance of ospC diversity for immune responses is well known [18]. However, to determine whether the genetic variation of subsections showing relationships with the reactivity difference would result in differences in protein expression, non-synonymous single-nucleotide polymorphisms (SNPs) were investigated using the “Find Variations/SNPs” tool in Geneious Prime 2023.1 (https://www.geneious.com/). The multiple nucleotide sequences and protein sequence alignments of the 23 ospC MGs were obtained using MAFFT v7.450 [27, 28] provided in Geneious. The ospC A sequence was set as the reference for calling SNPs and the possible effect on the protein due to the amino acid changes was provided by the “Find Variations/SNPs” tool following default settings.

## Results

### OspC MG phylogenetic relationship

**Fig 1:**
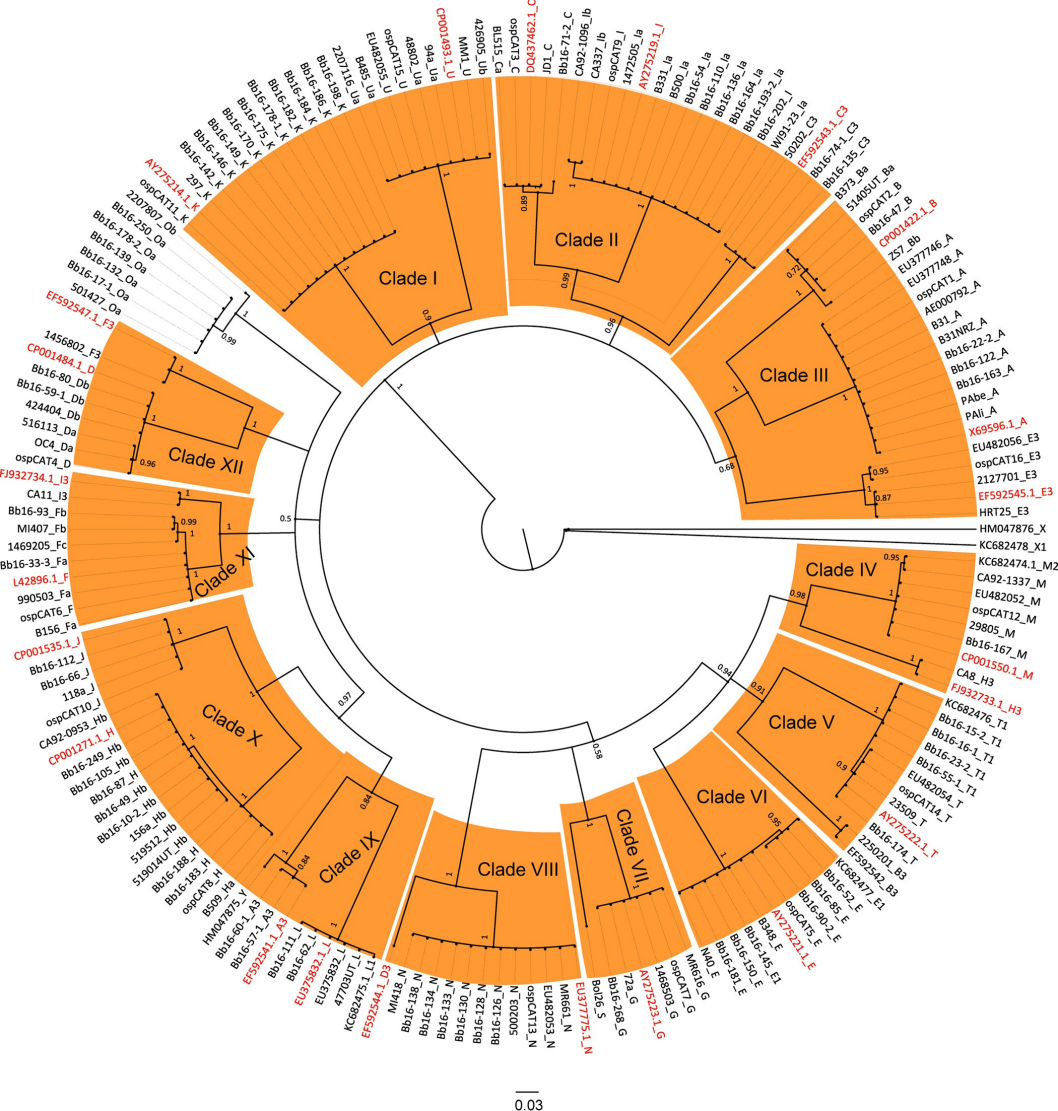
The maximum likelihood phylogenetic tree of nucleotide sequences of 47 ospC MGs and sub-types. The 23 ospC sequences used by Baum and colleagues [23] are highlighted in red. The clades gathering these sequences are numbered from I to XII and colored orange. ospC X (accession number: HM047876) was used as the outgroup. Sequences other than the 64 Canadian strains are named using NCBI accession number followed by the ospC MG type.

The global phylogenetic tree of the ospC MGs using 177 nucleotide sequences of 47 ospC MGs revealed that the 23 ospC MGs used by [23] are distributed within 12 monophyletic clades with high posterior probabilities covering the whole tree (Fig 1).

**Fig 2:**
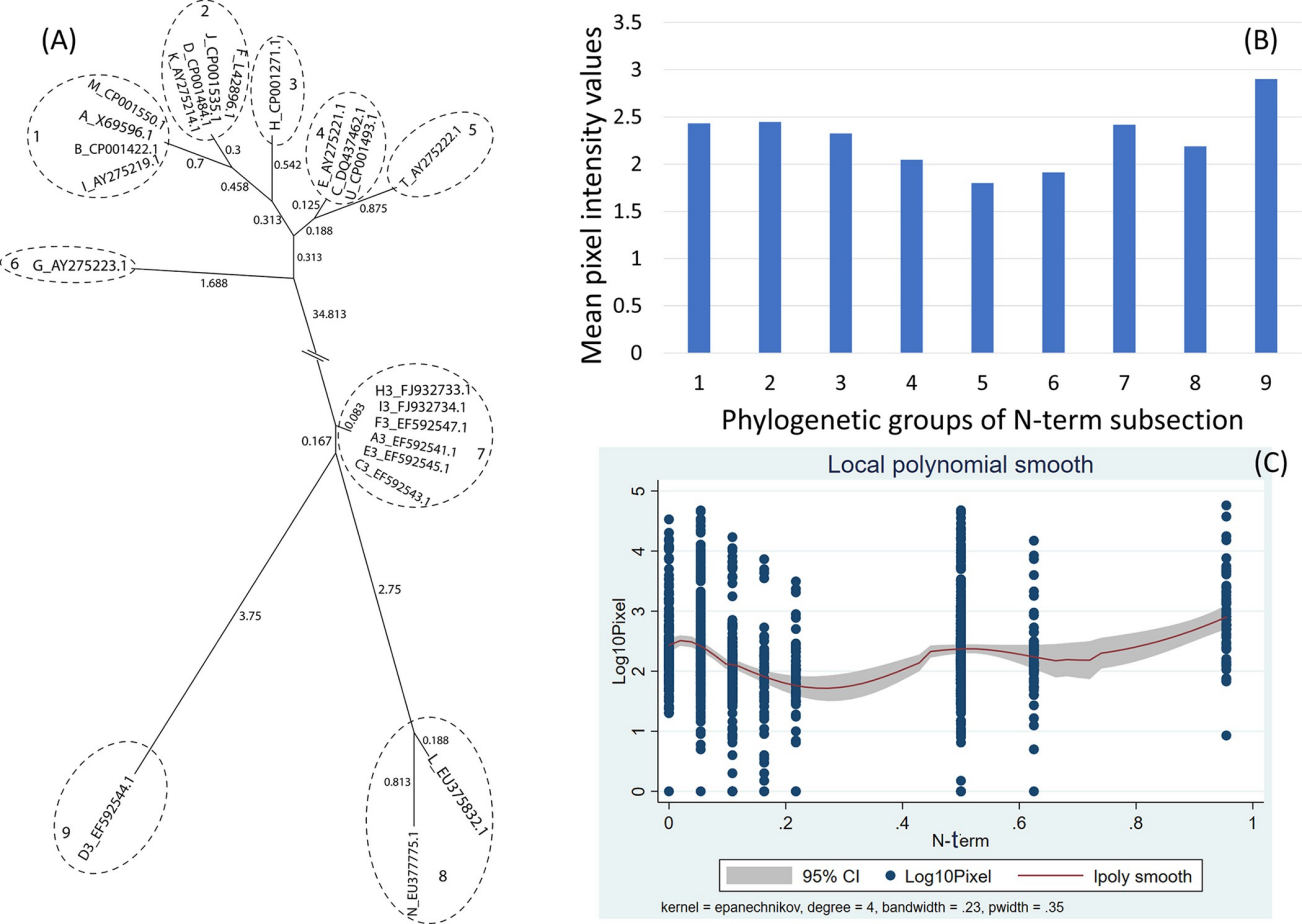
(A) Neighbor-Joining tree of the N-terminal fragment of 23 ospC MGs sequences built with near-minimal sum of branch-length estimated using Hamming distance. The phylogenetic groups were identified as numbers (1 to 9) and circled. (B) Histogram of the mean values of reactivity of each phylogenetic group and (C) the local polynomial smoothing of the polynomial model of the reactivity difference and genetic distance of the N-terminal fragment.

**Fig 3:**
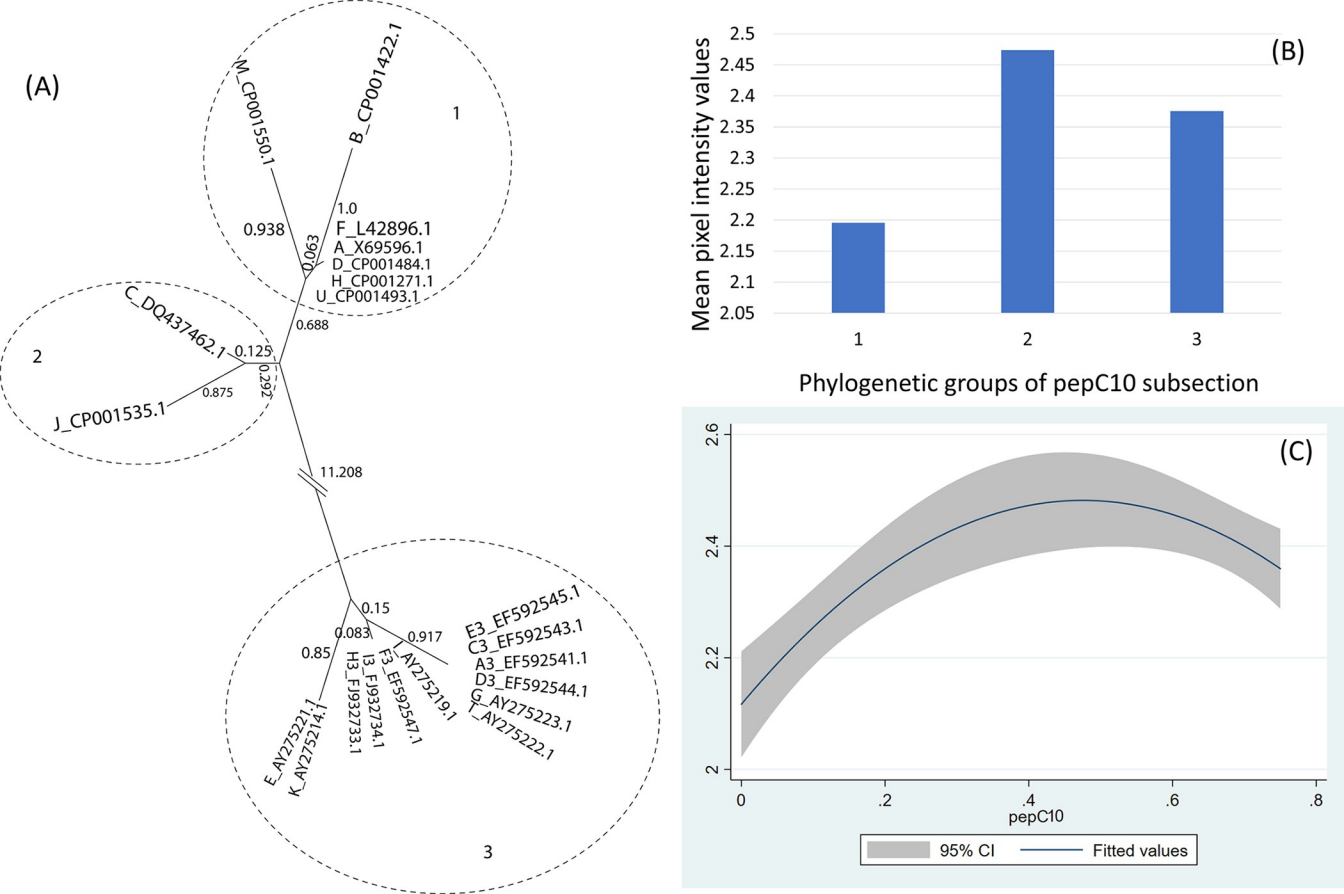
(A) Neighbor-Joining tree of the pepC10 fragment of 21 ospC MGs sequences built with near-minimal sum of branch-length estimated using Hamming distance. The phylogenetic groups were identified as numbers (1 to 3) and circled. (B) Histogram of the mean values of reactivity of each phylogenetic group and (C) the polynomial model of the reactivity difference and genetic distance of pepC10 fragment.

However, when restricting phylogenetic analysis of the 23 ospC MGs using only the sequences of the N-terminus and the highly conserved region of the C-terminus (i.e., pepC10), the analysis showed 9 phylogenetic groups for the first subsection (Fig 2A) and 3 phylogenetic groups for the second subsection (Fig 3A).

**Fig 4:**
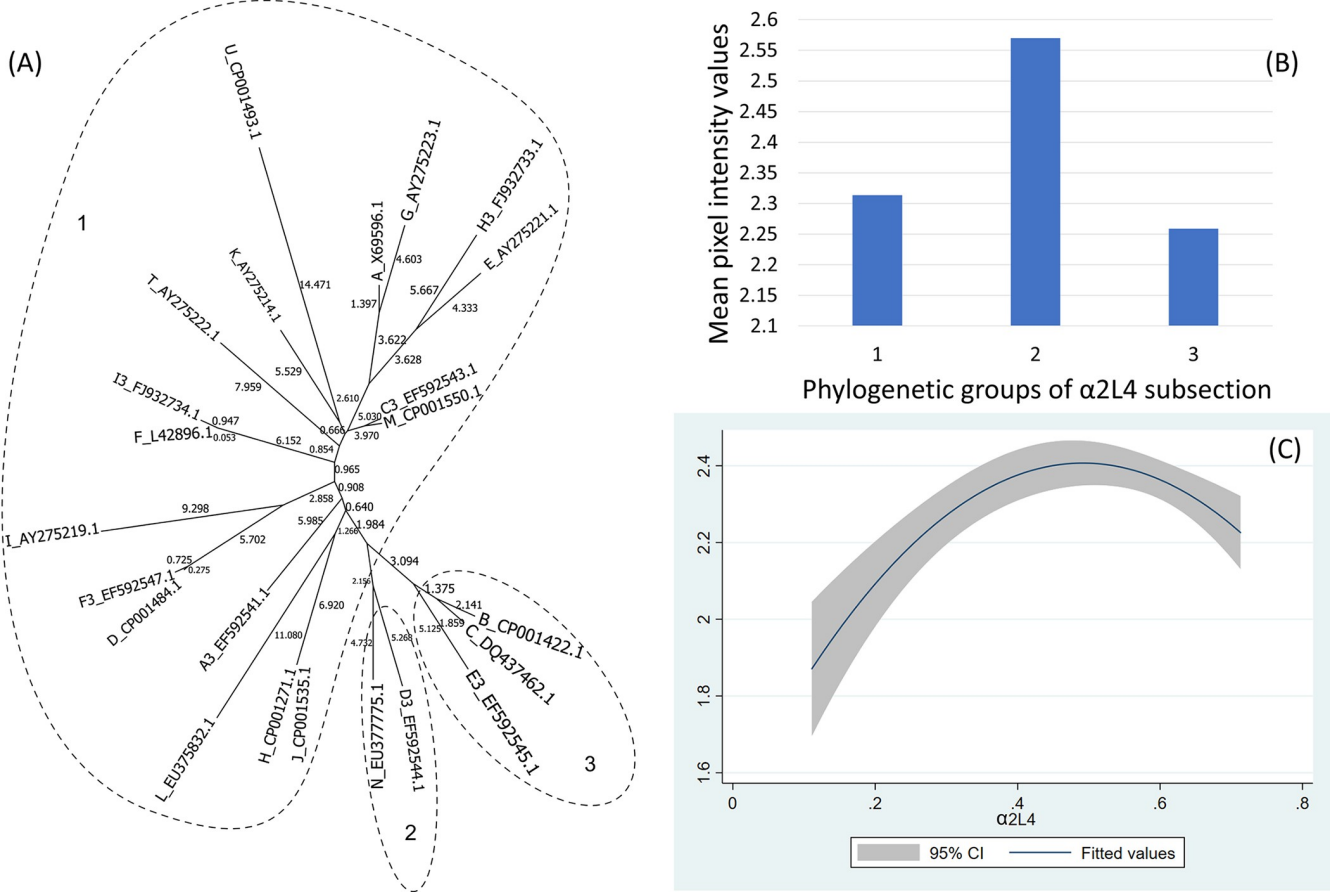
(A) Neighbor-Joining tree of the α2L4 fragment of 23 ospC MGs sequences built with near-minimal sum of branch-length estimated using Hamming distance. The phylogenetic groups were identified as numbers (1 to 3) and circled. (B) Histogram of the mean values of reactivity of each phylogenetic group and (C) the polynomial model of the reactivity difference and genetic distance of α2L4 fragment.

The α2L4 fragment is highly diverse showing 22 different genotypes where 5 ospC MGs form 2 clear clades corresponding to 2 phylogenetic groups, and 17 others (i.e., including the haplotype of ospC A) were gathered in the same phylogenetic group (Fig 4A).

**Fig 5:**
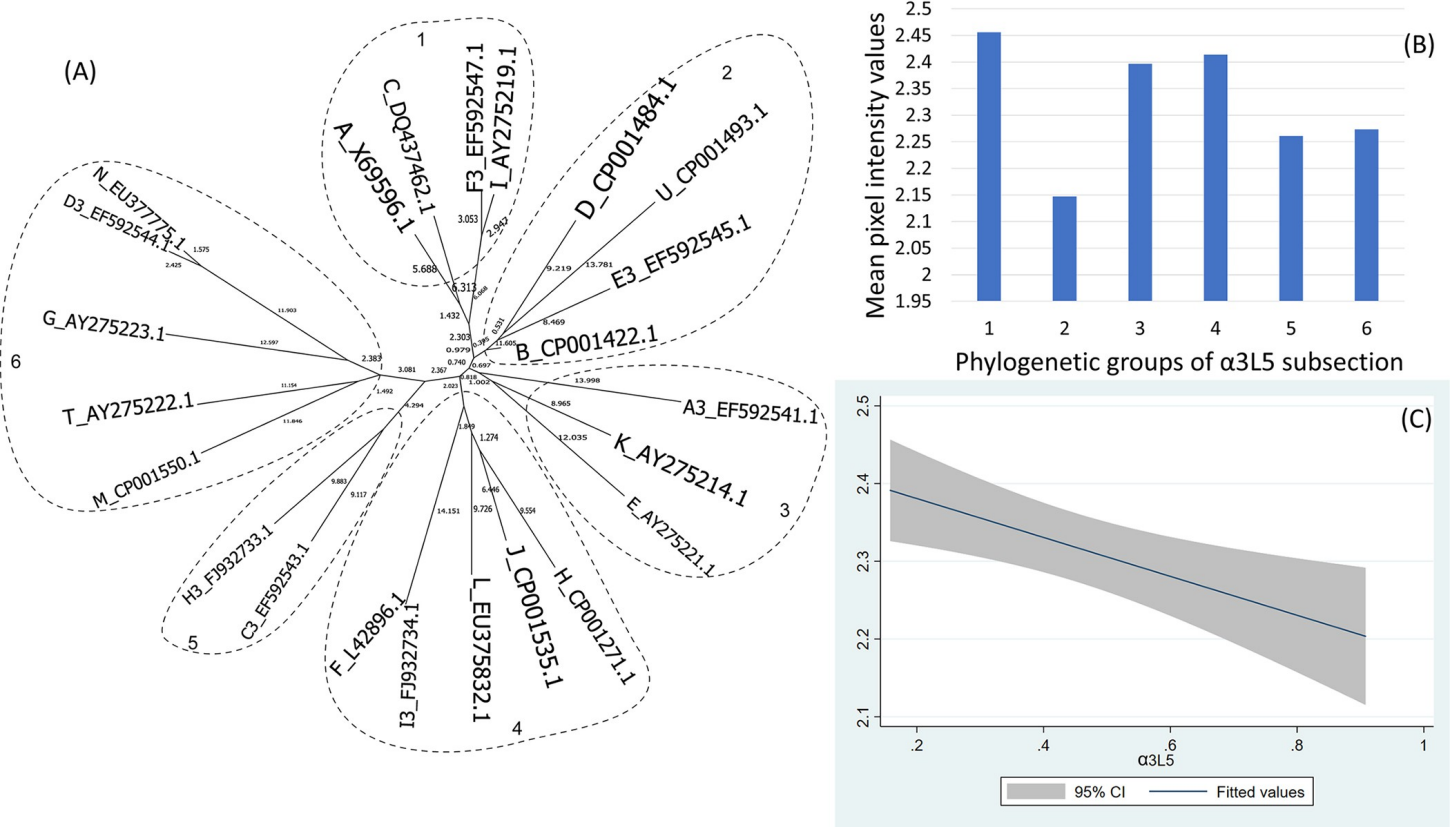
(A) Neighbor-Joining tree of the α3L5 fragment of 23 ospC MGs sequences built with near-minimal sum of branch-length estimated using Hamming distance. The phylogenetic groups were identified as numbers (1 to 6) and circled. (B) Histogram of the mean values of reactivity of each phylogenetic group and (C) the linear model of the reactivity difference and genetic distance of α3L5 fragment.

The α3L5 fragment is also highly diverse with 22 different genotypes which were clustered into 6 clear clades corresponding to 6 phylogenetic groups (Fig 5A).

**Fig 6:**
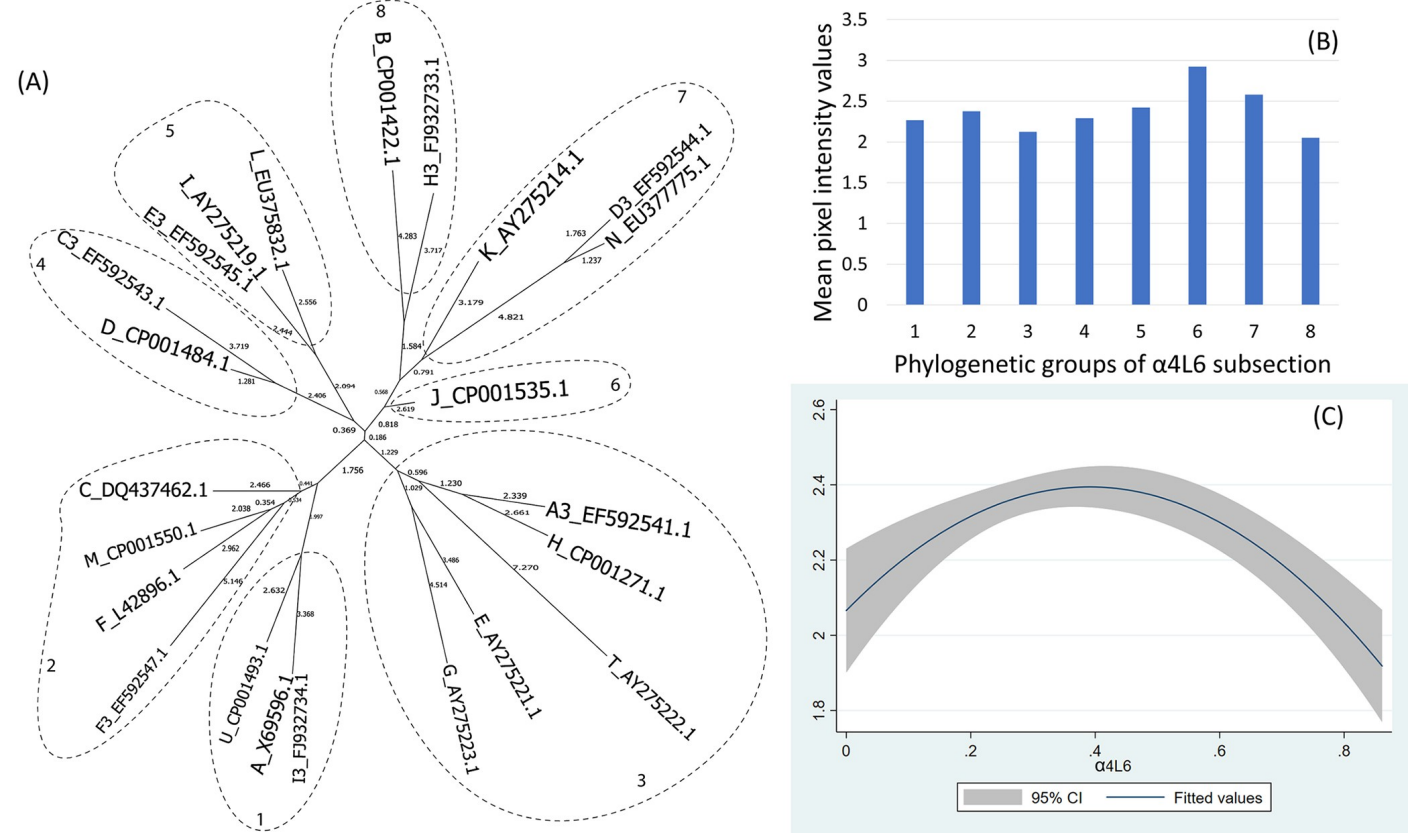
(A) Neighbor-Joining tree of the α4L6 fragment of 23 ospC MGs sequences built with near-minimal sum of branch-length estimated using Hamming distance. The phylogenetic groups were identified as numbers (1 to 8) and circled. (B) Histogram of the mean values of reactivity of each phylogenetic group and (C) the polynomial model of the reactivity difference and genetic distance of α4L6 fragment.

**Fig 7:**
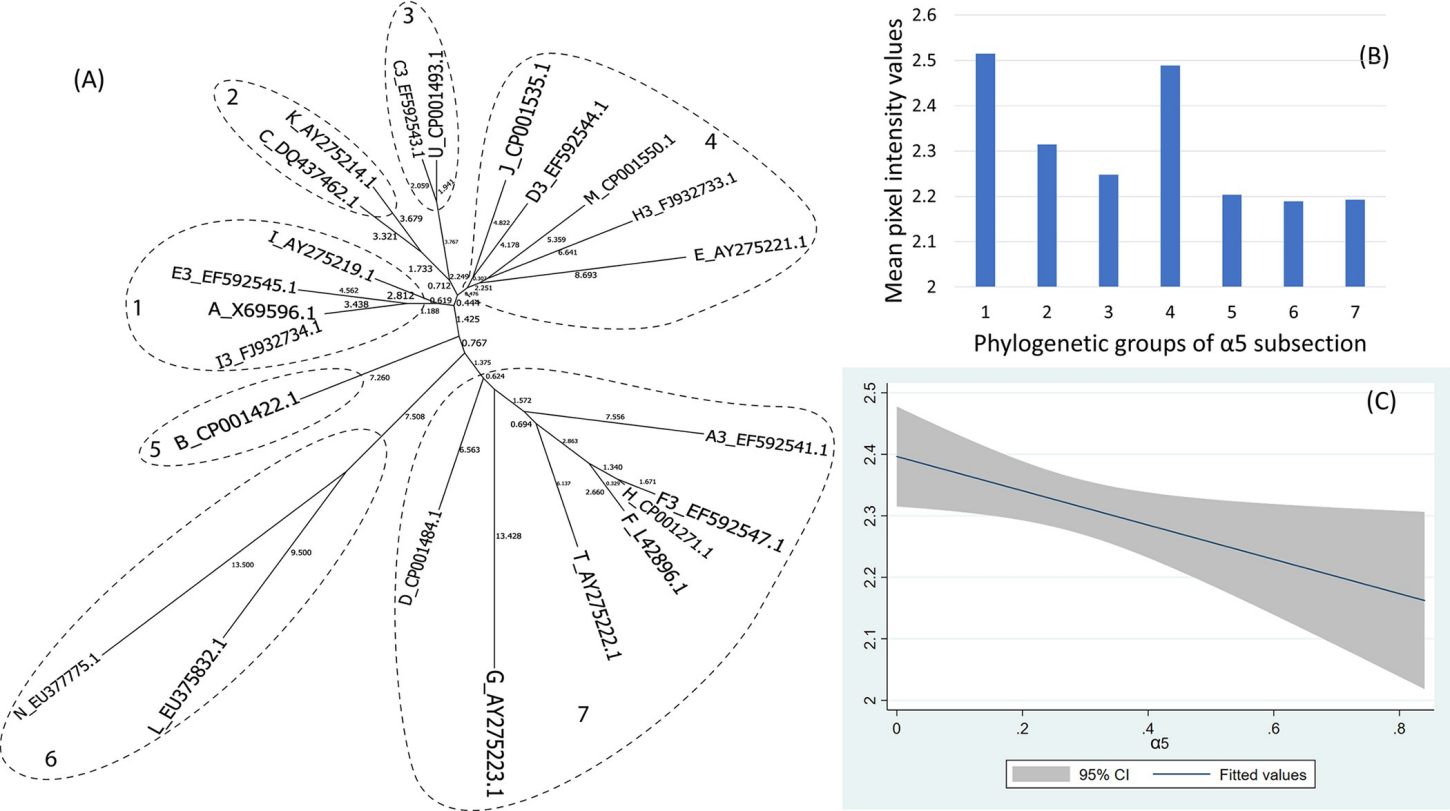
(A) Neighbor-Joining tree of the α5 fragment of 23 ospC MGs sequences built with near-minimal sum of branch-length estimated using Hamming distance. The phylogenetic groups were identified as numbers (1 to 7) and circled. (B) Histogram of the mean values of reactivity of each phylogenetic group and (C) the linear model of the reactivity difference and genetic distance of α5 fragment.

The α4L6 and α5 helices form, respectively, 8 and 7 phylogenetic groups (Figs 6A and 7A).

**Fig 8:**
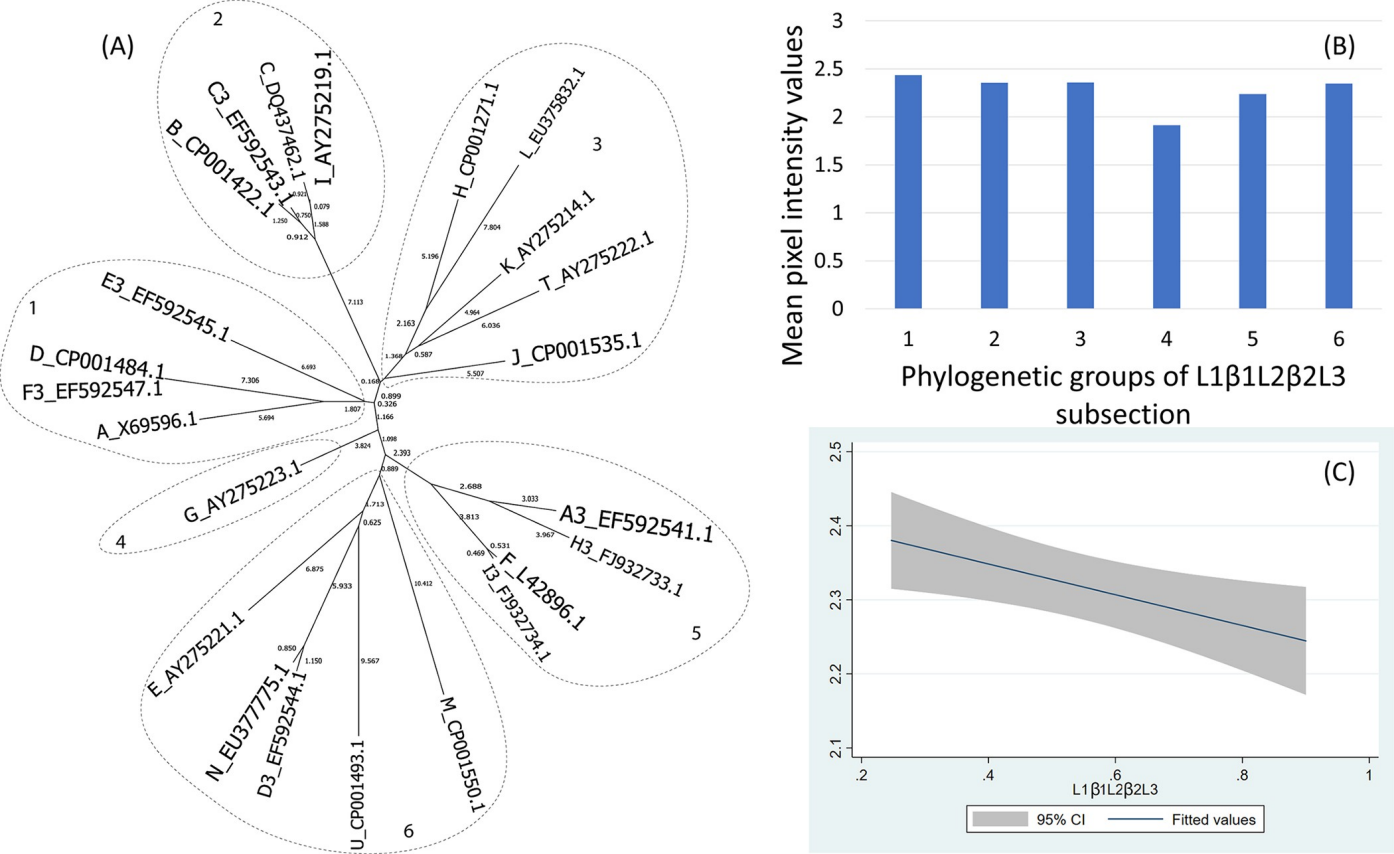
(A) Neighbor-Joining tree of the 2 β-strands fragment and loops L1-L2-L3 (L1β1L2β2L3) of 21 ospC MGs sequences built with near-minimal sum of branch-length estimated using Hamming distance. The phylogenetic groups were identified as numbers (1 to 9) and circled. (B) Histogram of the mean values of reactivity of each phylogenetic group and (C) the linear model of the reactivity difference and genetic distance of 2 β-strands fragment and loops L1-L2-L3.

The two β-strands connected by loops L1, L2, L3 (L1β1L2β2L3) created 6 phylogenetic groups forming distinct clades (Fig 8A).

### Statistical analysis

**Fig 9:**
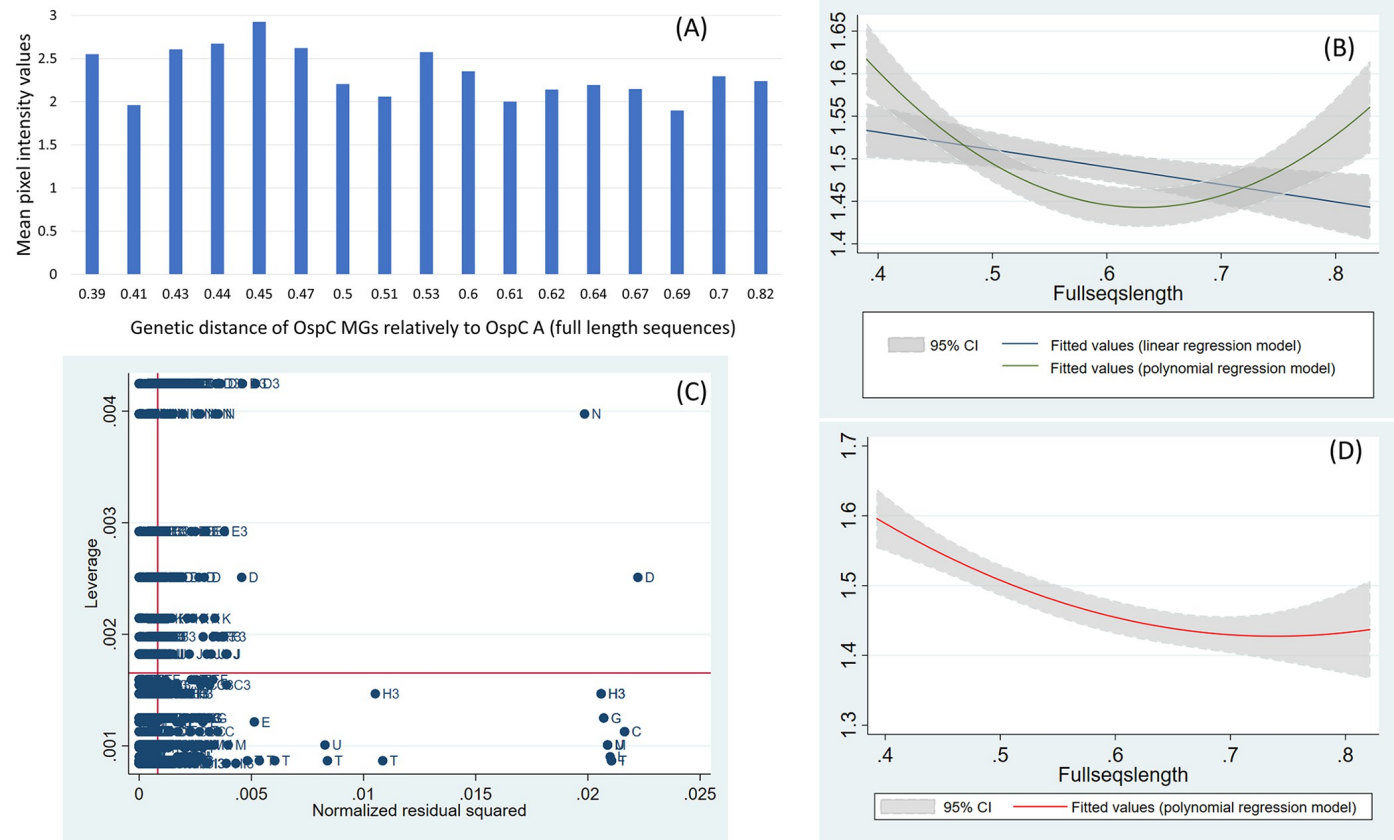
(A) Histogram of the mean values of reactivity and genetic distance of the full sequence length of 22 ospC MGs relative to ospC A. (B) Fitted values of linear and polynomial regression models of the reactivity difference and the genetic distance of 22 ospC MGs and ospC A. (C) Leverage versus squared residual plot for regression diagnostic. (D) Polynomial regression models of the reactivity difference and the genetic distance of 21 ospC MGs and ospC A, without data for ospC D3.

When investigating the relationship between the reactivity difference and the genetic distance of ospC A to the other 22 ospC MGs by linear regression, using the whole sequence length, there was a significant negative relationship (β = -0.52, CI = [-0.16, -0.88], P = 0.0047) (Table 1 in S1 File, Fig 9A), indicating that the increase of the genetic distance by 1 unit between ospC MGs and ospC A resulted in a reduction of the response signal by 14.33 units compared to the response to ospC A. However, the relationship was quadratic with significant polynomial terms (Table 2 in S1 File, Fig 9A). When visualizing the distribution of reactivity differences (Fig 9B), it is clear that the value for ospC D3 is responsible for this quadratic relationship (Fig 9C), and when repeating the analysis without the value for ospC D3, the relationship was negative and almost linear (Fig 9D). This suggests that in general, the genetic distance between ospC MGs and ospC A could have a significant role in the accuracy of the serological diagnostic test using ospC A as antigen.

**Fig 10:**
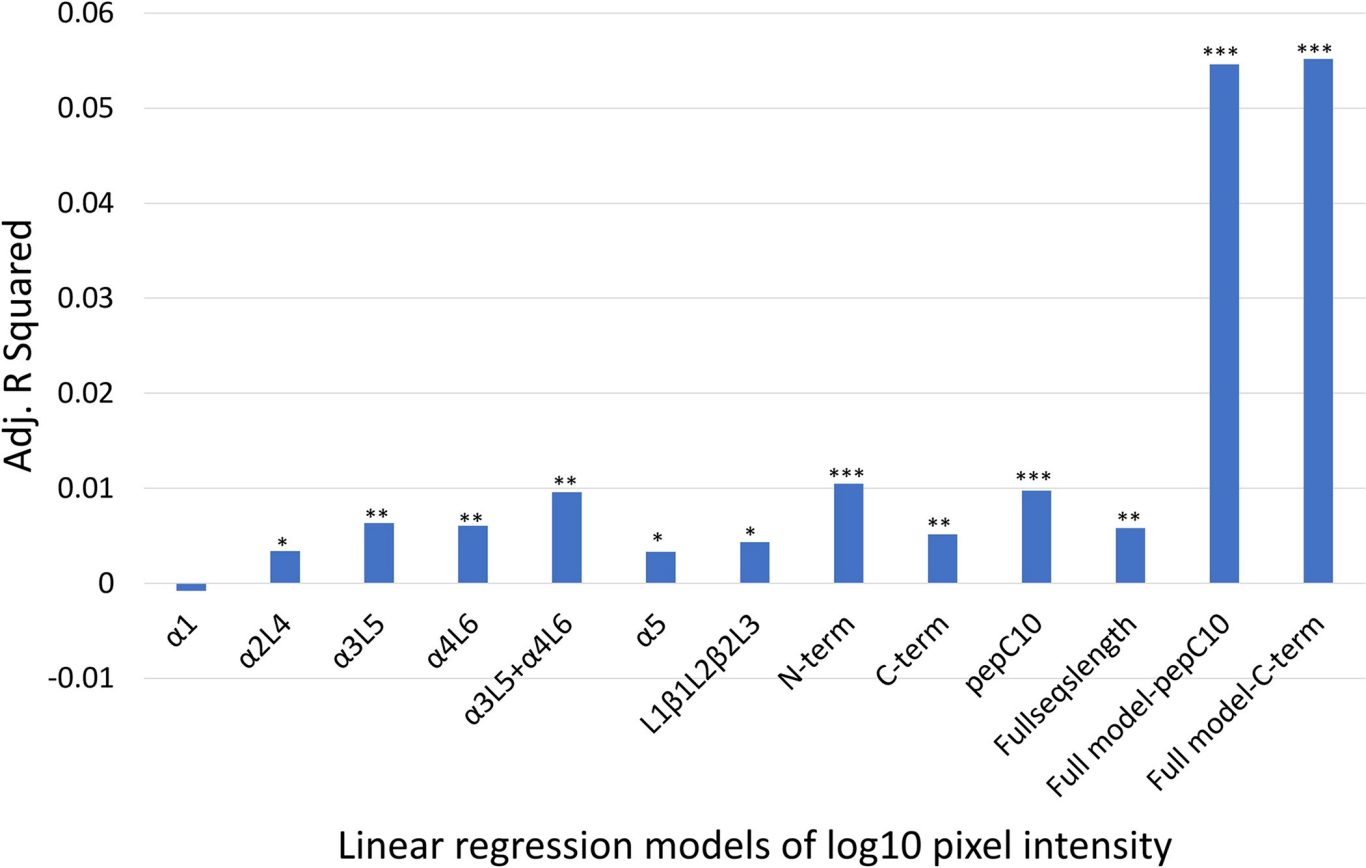
The adjusted R squared and the P value for the thirteen linear models that explored the relationship between genetic distance of ospC MGs from ospC A, and the reactivity difference. * = P < 0.05, ** = P < 0.001, *** = P < 0001.

To explore relationships between the reactivity difference and genetic distance of individual different subsections of the ospC protein sequences, 13 linear regression models were developed (10 simple linear models and 3 models with polynomial terms) (Fig 10). Except for the α1-helix model, which was not significant, all fragments had significant relationships between the reactivity difference and genetic distance.

For the N-terminus, there was a significant positive linear relationship with the reactivity difference but this relationship was complex (Fig 2B and 2C), with quadratic, cubic and quartic terms being significant (Table 3 in S1 File). Consequently, for this sequence, the relationship with reactivity difference was best explained by regression using the phylogenetic groups of the N-terminus as a categorical explanatory variable (Fig 2A). In this analysis, phylogenetic groups 4, 5, 6 and 9 were negatively related to the reactivity difference compared to those of group 1 that includes the ospC A MG (Table 4 in S1 File).

For the C-terminus and pepC10 fragments, here was a significant quadratic relationship between genetic distance and reactivity difference (Table 5 in S1 File, Fig 3B and 3C) (only pepC10 results are displayed because the C-terminus showed a similar result). The negative association of the quadratic term was associated with the genetic distance between phylogenetic group 2 and 3 (Fig 3A, Table 6 in S1 File). A comparable concave relationship was observed for the second α helix linked with loop 4 (α2L4) (Fig 4B and 4C) where polynomial terms are significant (Table 7 in S1 File), and in categorical analysis there were significant differences between phylogenetic groups 2 and 3 (β = -0.31, CI = [-0.50, -0.12], P = 0.000) (Table 8 in S1 File). For fragments α3L5 (Table 9 in S1 File, Fig 5B and 5C), α5 (Table 10 in S1 File, Fig 7B and 7C) and the 2 β-strands connected by 3 loops (L1, L2, L3) (Table 11 in S1 File, Fig 8B and 8C) the relationships between genetic distance and reactivity difference were significant, linear and negative. For the α4L6 fragment, the relationship was more concave (Fig 6B and 6C) and the second polynomial term was significant (Table 12 in S1 File). In categorical analysis, there were significant differences between groups 1 and 8 (Table 13 in S1 File).

### Single-nucleotide polymorphism detection

In the analysis of SNPs in the subsections corresponding to the three fragments showing a linear relationship between genetic distance and reactivity difference (α3L5, α5 and L1β1L2β2L3) there were 10 SNPs (Fig 1 in S1 File) including 9 substitutions which cause amino acid changes relative to the OspC A sequence and 1 deletion (located at position 253 corresponding to position 85 of the protein MSA, Fig 1 in S1 File), which causes a frame shift of the β1 strand. The ospC MGs L (clade 3), A3, H3, I3, F (clade 5), and U (clade 6) had a substitution coding for arginine compared to glutamine in OspC A (at position 83 of the protein MSA), while for MGs of clades 3 (K, T, H, J), 5 (H3) and 6 (U) SNPs resulted in coding for glycine rather than asparagine in OspC A (at position 92 of the protein MSA, Fig 1 in S1 File). Another SNP detected on the L1β1L2β2L3 fragment at position 91 coded for arginine rather than asparagine in OspC A. The ospC MGs D, E3 and U of clade 2 of the α3L5 fragment had 2 substitutions, replacing glycine by asparagine and lysine by serine; both mutations occur also for the more distant clades from clade 1 containing ospC A: clades 3 (K, E, A3), 4 (J), 5 (C3, H3) and 6 (G, M, T) (Fig 1 in S1 File). For members of clades 3 (E), 4 (L), 5 (C3, H3) and 6 (T, M) glutamate in OspC A is replaced by serine (Fig 1 in S1 File). Two other substitutions, alanine to lysine (at position 130) and threonine to glutamate (at position 137) were found in all clades, except the clade 5. For the α5 fragment, only one substitution was detected (i.e., glycine replaced by lysine at position 181), which concerns six of the seven members of clade 7, clade 6 (L), and clade 4 (J) (Fig 1 in S1 File).

## Discussion

The outer surface protein C is a major virulence factor of *B*. *burgdorferi*, and it’s expression is required to establish infection in a mammalian host after a tick bite [36, 37]. The expression of OspC is up-regulated during the infection process then subsequently down-regulated [38, 39]. Accumulating mutations in surface-exposed regions of the OspC protein [40] may be one strategy used by *B*. *burgdorferi* to escape the host immune system [41]. This inter-strain antigenic variation could have an impact on the performance of serological diagnostic tests, because antibodies raised against a specific infecting strain could bind with lower affinity to antigens of strains that are genetically different [22]. Therefore, in this study we hypothesized that the sensitivity of the serological test decreases with the genetic distance of infecting strains to the B31 strain. We tested this hypothesis by modeling the difference in intensity of serological responses of an infected population to 22 ospC major group sequences, compared to the intensity of responses to ospC major group A, as a function of the genetic distance between the ospC major groups and ospC A. The intensity of the antibody response was investigated previously by Baum and colleagues [23] to explore the cross-reactivity between the 23 ospC MGs (i.e., Pearson’s r correlation), which revealed differences between certain ospC variants (e.g., ospC A versus ospC H3, G, A3, N, K and J had showed a Pearson’s r ≤ 0.66). However, for Baum and colleagues [23], the highly correlated ospC variants are the most cross-reactive and the identity of the amino acid sequences of the fifth C-terminal helix was a significant explanatory factor of this type-specific cross-reactivity.

We demonstrated in this in-silico study that there is a significant association between the genetic diversity of the ospC gene and the difference in immunoreactivity to OspC A and to other OspC MG proteins within an infected population who had been diagnosed using serological assays that employed OspC A as antigen; i.e. the null hypothesis of statistical models was rejected. This result is consistent with Di and colleagues [19], which reveled that *B*. *burgdorferi* strains carrying different ospC MGs naturally co-infecting the same host displayed immunological distinctness, which means that in natural infections, the host immune system react differently to different ospC MGs. However, by an in-depth investigation of the relationship between local nucleotide sequence identity and antibody response intensity, it appears that this relationship is more complex for the subsection sequences mostly used to design epitopes targeted during the two-step EIA and the EIA-WB two-tier diagnostic tests (i.e., N-terminus, C-terminus and pepC10 of the C-terminal region) [42]. These regions of OspC showed a non-linear relationship between reactivity difference and genetic distance (i.e., sine curve shape for the N-terminus and concave parabola curve shape for pepC10). These complex relationships were better explained by clade membership in the NJ phylogenetic trees of these sequences where some, but not all, ospC MGs in clades distant from that containing B31 had lower reactivity relative to the phylogenetic clade in which ospC A occurs. This finding supports that to some degree the more distant an ospC MG is to ospC A, the weaker the antibody signal response is, but this relationship is complex and not fully explained simply by genetic distance. Other factors may interact in the antibody binding process among the ospC variants. Experimental studies have reported that mutations (e.g., deletions) occurring in the N- and C-terminus regions led to the abolition of the binding of monoclonal antibodies against OspC [43, 44], supporting at least one role that genetic variation may have in affecting OspC immunoreactivity. Theison and colleagues [45] demonstrated that the epitope diversity of OspC is reflected in serotype-restricted specificity. Our results are coherent with previous studies [20, 46, 47] reporting that despite strong sequence conservation in the N- and C-terminus of OspC, their antibody cross-reactivity remains type specific. In contrast, Baum and colleagues [23] indicated that the global similarity between OspC amino acid sequences was a poor predictor of the cross-reactive antibody binding except for the C-terminus region. As a difference with [23], we investigated the nucleotide sequence similarity rather than amino acid identity. DNA sequences are known to be often more informative than amino acid sequences in terms of detecting phylogenetic patterns because they have more characters and synonymous as well as non-synonymous substitutions (the latter not impacting amino acid coding) that may signal evolutionary changes, which makes them able to better capture any evolutionary patterns [48].

Investigating regions of the OspC protein other than the extremities allowed identification of three zones significantly and linearly associated with the decrease of the intensity signal of the antibody response of OspC: the α-helices, 3 (connected by the loop 5) and 5, and the 2 β-strands (connected by loops 1, 2, 3). The α-helix 3, with helix 4, forms a lattice zone on the protein surface which might be involved in protein-protein or protein-ligand binding [49] allowing it to be a possible region of interest for antibody binding. The α-helix 5 is known to be highly variable between ospC types, but highly conserved within a type which offers a promising solution for developing a chimeric OspC vaccinogen product [50]. This large helix is located within a surface-exposed domain [51] and an earlier study demonstrated immunogenicity of this region for ospC types A, B, K and D [52]. Both small β-strands connected by three loops (1, 2 and 3) are also surface-exposed [53] and known to be involved in the stabilization of OspC dimers [49]. Deletions occurring within this region had been shown to result in a complete loss of infectivity [54]. Its great stability due in particular to its functional role [55] has made it a good candidate antigen for diagnostics, but there was sufficient variability impacting the reactivity of sera from infected people. Interestingly, Ivanova and colleagues [20] demonstrated that epitopes of OspC types B, E, F and K may allow increased sensitivity of serodiagnostic assays for detecting most anti-OspC antibodies present in serum samples from seropositive patients infected with *B*. *burgdorferi*. Indeed, in trees constructed for the three subsections L1β1L2β2L3, α3L5 and α5, the B, E, F and K ospC MGs occur within the most distant clades to ospC A, and perhaps including MGs from these clades could be considered in the development of serodiagnosis using OspC antigen. Di and colleagues [19] proposed four centroids of OspC antigen to obtain a broader antigenicity, because they were derived from 16 native ospC MGs. However, after aligning the sequences of these centroids with the 23 sequences used by Baum and colleagues [23] and generating an ML tree (data not shown), it appeared that all the centroids form the same phylogenetic group (FastTree supporting values of 89%) with only 13 other ospC MGs (i.e., E, D3, N, B, E3, C3, I, C, K, A, U, M, and H3). This could indicate that the four centroids are not representative of the total variation known for the OspC antigen, whereas the 23 sequences used here seemed to be well distributed within the 47 OspC MGs presented in the global tree. Clearly though, the observations of associations between genetic distance and cross-reactivity with the OspC A antigen need to be confirmed by further well designed experimental studies.

The investigation of single nucleotide polymorphisms (SNPs) in this study identified nine substitutions and one deletion within three fragments showing a linear relationship between genetic distance and reactivity difference. This supports the idea that exploring phylogeny of the ospC MGs (by genetic distance and clade structure) is meaningful in terms of amino acid changes that can cause conformational modifications in the protein structure or physicochemical alterations that could affect antibody binding in vitro and in vivo [56]. For example, the change of glutamine to alanine in L1β1L2β2L3 subsection (at 83 position of the MSA) occurring within all members of clade 5 in Fig 1 (S1 File) (i.e., A3, H3, I3, F), which is distant from clade 1 in which ospC A occurs, may disrupt hydrogen bonding interactions and potentially altering the local protein structure. This is because glutamine is a polar aa with a side chain that can form hydrogen bonds, while alanine is nonpolar and lacks a similar side chain [57]. Therefore, this substitution may disrupt hydrogen bonding interactions and potentially altering the local protein structure.

The deletion in the β-strand 1 at position 85 resulting in removal of an asparagine amino acid recontributing to the distinction of three clades (4, 5, 6) from clade 1 of ospC A within the L1β1L2β2L3 fragment. Asparagine residues are often found in protein structural subsections and can participate in hydrogen bonding interactions that help stabilize the protein’s secondary structure, including β-strands [58]. The removal of asparagine can disrupt these interactions, leading to alterations in the protein’s overall fold [57]. In the case of the α3L5 fragment, two replacements of glutamate (at 144 position) and lysine (at 145 position), both to serine were detected and are associated with the clade 5 (i.e., C3, H3) of this subsection. Glutamate is a negatively charged aa, while lysine is positively charged, and serine is neutral [57]. The substitution of charged amino acids with a neutral one results in a net charge change in the α3L5, potentially impacting its interactions with other molecules or proteins that rely on charge-based interactions [56]. Other non-synonymous changes had similar impacts on phylogeny and likely similar impacts on functional protein structure and interactions. It is very possible that protein changes associated with non-synonymous SNPs would not have simplistic impacts on antibody binding, and this could be partly responsible for the more complex relationships between genetic distance and reactivity. In this in silico study, we highlight the possible role that genetic diversity of *B*. *burgdorferi*, and genetic distance from the antigen used in diagnostic kits, can play in the serodiagnosis of Lyme disease when this is based on the OspC antigen. However, this observation needs to be confirmed using experimental studies. Further prospective investigations are needed to investigate strain-diversity-related factors that affect antibody binding of ospC MGs, and any consequent impacts on performance of serodiagnostic assays for Lyme disease.

## Supporting information

S1 File (DOCX)
