# Supplementary material for: Possible effect of mutations on serological detection of Borrelia burgdorferi sensu stricto ospC major groups: An in-silico study
Source: PLoS One. 2023 Oct 10;18(10):e0292741. doi: 10.1371/journal.pone.0292741 (PMC10564231; doi:10.1371/journal.pone.0292741)
Supplement: S1 File — (DOCX) [file pone.0292741.s001.docx]

**Supplemental information**

**Title:** Possible effect of mutations on serological detection of *Borrelia burgdorferi* sensu stricto ospC major groups: an in-silico study.

**Authors:** Samir Mechai, Heather Coatsworth and Nicholas H. Ogden

**Tables**

**Table 1.** Linear regression model of the log10 pixel intensity signal using the genetic distance of the full sequence of the 22 ospC MGs. The 95% of CI is reported in the table.

| Variable | Coefficient | Std Error | t Ratio | P Value | Conf. High | Conf. Low |
| --- | --- | --- | --- | --- | --- | --- |
| (Intercept) | 2.62 | 0.11 | 24.00 | 0.000 | 2.84 | 2.41 |
| Fullseqslength | -0.52 | 0.18 | -2.84 | 0.0047 | -0.16 | -0.88 |

**Table 2.** Quadratic polynomial regression model of the log10 pixel intensity signal as dependent variable using the genetic distance of the full sequence length. The 95% of CI is reported in the table.

| Variable | Coefficient | Std Error | t Ratio | P-Value | Conf. High | Conf. Low |
| --- | --- | --- | --- | --- | --- | --- |
| (Intercept) | 2.63 | 0.18 | 14.94 | 0.000 | 2.29 | 2.98 |
| Fullseqslength | -3.77 | 0.60 | -6.26 | 0.000 | -4.96 | -2.59 |
| Fullseqslength ^2^ | 2.99 | 0.50 | 5.96 | 0.000 | 2.00 | 3.97 |

**Table 3.** Quartic polynomial regression model of the log10 pixel intensity signal as dependent variable using the N-Term genetic distance. The 95% of CI is reported in the table.

| Variable | Coefficient | Std Error | t Ratio | P-Value | Conf. High | Conf. Low |
| --- | --- | --- | --- | --- | --- | --- |
| (Intercept) | 2.56 | 0.05 | 48.00 | 0.000 | 2.46 | 2.67 |
| N-Term | -6.97 | 1.19 | -5.85 | 0.000 | 9.31 | -4.63 |
| N-Term^2^ | 28.98 | 7.17 | 4.04 | 0.000 | 14.92 | 43.03 |
| N-Term^3^ | -42.27 | 14.52 | -2.91 | 0.004 | 70.75 | -13.78 |
| N-Term^4^ | 20.89 | 8.65 | 2.42 | 0.016 | 3.92 | 37.86 |

**Table 4.** Linear regression model of the log10 pixel intensity signal as dependent variable using the N-Term phylogenetic groups as independent variables (group 1 is the base level containing the N-term of ospC A and other identical haplotypes). The 95% of CI is reported in the table.

| Variable | Coefficient | Std Error | t Ratio | P-Value | Conf. High | Conf. Low | VIF | Base Level |
| --- | --- | --- | --- | --- | --- | --- | --- | --- |
| (Intercept) | 2.43 | 0.06 | 41.84 | 0.000 | 2.32 | 2.55 |  |  |
| Phylogenetic groups |  |  |  |  |  |  |  |  |
| 2 | 0.02 | 0.08 | 0.21 | 0.834 | -0.13 | 0.17 | 1.91 | 1 |
| 3 | -0.10 | 0.12 | -0.9 | 0.367 | -0.33 | 0.12 | 1.27 | 1 |
| 4 | -0.40 | 0.08 | -4.84 | 0.000 | -0.56 | -0.24 | 1.73 | 1 |
| 5 | -0.63 | 0.12 | -5.43 | 0.000 | -0.86 | -0.40 | 1.27 | 1 |
| 6 | -0.52 | 0.12 | -4.45 | 0.000 | -0.75 | -0.29 | 1.27 | 1 |
| 7 | -0.01 | 0.07 | -0.19 | 0.850 | -0.15 | 0.13 | 2.18 | 1 |
| 8 | 0.47 | 0.12 | 4.05 | 0.000 | 0.24 | 0.70 | 1.27 | 1 |
| 9 | -0.24 | 0.09 | -2.63 | 0.009 | -0.42 | -0.06 | 1.52 | 1 |

**Table 5.** Cubic polynomial regression model of the log10 pixel intensity signal as dependent variable using pepC10 genetic distance. The 95% of CI is reported in the table.

| Variable | Coefficient | Std Error | t Ratio | P-Value | Conf. High | Conf. Low |
| --- | --- | --- | --- | --- | --- | --- |
| (Intercept) | 2.09 | 0.05 | 40.35 | 0.000 | 1.99 | 2.19 |
| pepC10 | 2.74 | 0.83 | 3.3 | 0.001 | 1.11 | 4.36 |
| pepC10^2^ | -5.94 | 2.77 | -2.15 | 0.032 | -11.37 | -0.52 |
| pepC10^3^ | 3.73 | 2.36 | 1.58 | 0.114 | -0.90 | 8.35 |

**Table 6.** Linear regression model of the log10 pixel intensity signal as dependent variable using the pepC10 phylogenetic groups as independent variables. The 95% of CI is reported in the table.

| *Variable* | *Coefficient* | *Std Error* | *t Ratio* | *P-Value* | *Conf. High* | *Conf. Low* | *VIF* | *Base Level* |
| --- | --- | --- | --- | --- | --- | --- | --- | --- |
| *(Intercept)* | 2.47 | 0.07 | 33.39 | 0.000 | 2.33 | 2.62 |  |  |
| Phylogroups of pepC10 |  |  |  |  |  |  |  |  |
| 1 | -0.23 | 0.08 | -2.71 | 0.007 | -0.39 | -0.06 | 3 | 2 |
| 3 | -0.10 | 0.08 | -1.23 | 0.220 | -0.26 | 0.06 | 3 | 2 |

**Table 7.** Quadratic polynomial regression model of the log10 pixel intensity signal as dependent variable using genetic distance of the α2L4 fragment. The 95% of CI is reported in the table.

| *Variable* | *Coefficient* | *Std Error* | *t Ratio* | *P-Value* | *Conf. High* | *Conf. Low* |
| --- | --- | --- | --- | --- | --- | --- |
| *(Intercept)* | 1.51 | 0.14 | 10.08 | 0.000 | 1.21 | 1.8 |
| α2L4 | 3.63 | 0.68 | 5.33 | 0.000 | 2.30 | 4.97 |
| α2L4^2^ | -3.69 | 0.74 | -4.96 | 0.000 | -5.16 | -2.23 |

**Table 8.** Linear regression model of the log10 pixel intensity signal as dependent variable using the α2L4 fragment phylogenetic groups as independent variables (group 1 is the base level containing the α2L4 fragment of ospC A and other identical or closest haplotypes). The 95% of CI is reported in the table.

| *Variable* | *Coefficient* | *Std Error* | *t Ratio* | *P-Value* | *Conf. High* | *Conf. Low* | *VIF* | *Base Level* |
| --- | --- | --- | --- | --- | --- | --- | --- | --- |
| *(Intercept)* | 2.57 | 0.07 | 34.70 | 0.00 | 2.42 | 2.72 |  |  |
| Phylogroups of α2L4 |  |  |  |  |  |  |  |  |
| *1* | -0.26 | 0.08 | -3.28 | 0.00 | -0.41 | -0.10 | 2.17 | 2 |
| *3* | -0.31 | 0.10 | -3.25 | 0.00 | -0.50 | -0.12 | 2.17 | 2 |

**Table 9.** Linear regression model of the log10 pixel intensity signal using the genetic distance of the α3L5 fragment as independent variable. The 95% of CI is reported in the table.

| *Variable* | *Coefficient* | *Std Error* | *t Ratio* | *P-Value* | *Conf. High* | *Conf. Low* |
| --- | --- | --- | --- | --- | --- | --- |
| *(Intercept)* | 2.43 | 0.04 | 55.14 | 0.000 | 2.34 | 2.52 |
| a3L5 | -0.25 | 0.08 | -2.95 | 0.003 | -0.42 | -0.08 |

**Table 10.** Linear regression model of the log10 pixel intensity signal using the genetic distance of the α5 fragment as independent variable. The 95% of CI is reported in the table.

| *Variable* | *Coefficient* | *Std Error* | *t Ratio* | *P-Value* | *Conf. High* | *Conf. Low* |
| --- | --- | --- | --- | --- | --- | --- |
| *(Intercept)* | 2.40 | 0.04 | 57.97 | 0.000 | 2.32 | 2.48 |
| α5 | -0.28 | 0.12 | -2.24 | 0.025 | -0.52 | -0.03 |

**Table 11.** Linear regression model of the log10 pixel intensity signal using the genetic distance of the L1B1L2B2L3 fragment as independent variable. The 95% of CI is reported in the table.

| *Variable* | *Coefficient* | *Std Error* | *t Ratio* | *P-Value* | *Conf. High* | *Conf. Low* |
| --- | --- | --- | --- | --- | --- | --- |
| *(Intercept)* | 1.54 | 0.02 | 80.56 | 0.000 | 1.50 | 1.57 |
| L1B1L2B2L3 | -0.08 | 0.03 | -2.56 | 0.011 | -0.14 | -0.02 |

**Table 12.** Quadratic polynomial regression model of the log10 pixel intensity signal as dependent variable using genetic distance of the α4L6 fragment. The 95% of CI is reported in the table.

| *Variable* | *Coefficient* | *Std Error* | *t Ratio* | *P-Value* | *Conf. High* | *Conf. Low* |
| --- | --- | --- | --- | --- | --- | --- |
| *(Intercept)* | 1.68 | 0.39 | 4.33 | 0.000 | 0.92 | 2.45 |
| α4L6 | 1.68 | 0.39 | 4.33 | 0.000 | 0.92 | 2.45 |
| α4L6^2^ | -2.15 | 0.39 | -5.49 | 0.000 | -2.92 | -1.38 |

**Table 13.** Linear regression model of the log10 pixel intensity signal as dependent variable using the α4L6 fragment phylogenetic groups as independent variables (group 1 is the base level containing the α4L6 fragment of ospC A and other identical or closest haplotypes). The 95% of CI is reported in the table.

| *Variable* | *Coefficient* | *Std Error* | *t Ratio* | *P-Value* | *Conf. High* | *Conf. Low* | VIF | *Base Level* |
| --- | --- | --- | --- | --- | --- | --- | --- | --- |
| *(Intercept)* | 2.27 | 0.06 | 38.62 | 0.00 | 2.15 | 2.38 |  |  |
| Phylogroups of α4L6 |  |  |  |  |  |  |  |  |
| 2 | 0.11 | 0.08 | 1.38 | 0.17 | -0.05 | 0.26 | 1.93 | 1 |
| 3 | -0.14 | 0.07 | -1.94 | 0.05 | -0.29 | 0.00 | 2.09 | 1 |
| 4 | 0.02 | 0.09 | 0.26 | 0.80 | -0.16 | 0.21 | 1.52 | 1 |
| 5 | 0.15 | 0.08 | 1.85 | 0.07 | -0.01 | 0.32 | 1.74 | 1 |
| 6 | 0.66 | 0.12 | 5.59 | 0.00 | 0.43 | 0.89 | 1.28 | 1 |
| 7 | 0.31 | 0.08 | 3.77 | 0.00 | 0.15 | 0.48 | 1.74 | 1 |
| 8 | -0.22 | 0.09 | -2.34 | 0.02 | -0.40 | -0.04 | 1.52 | 1 |

**Figures**


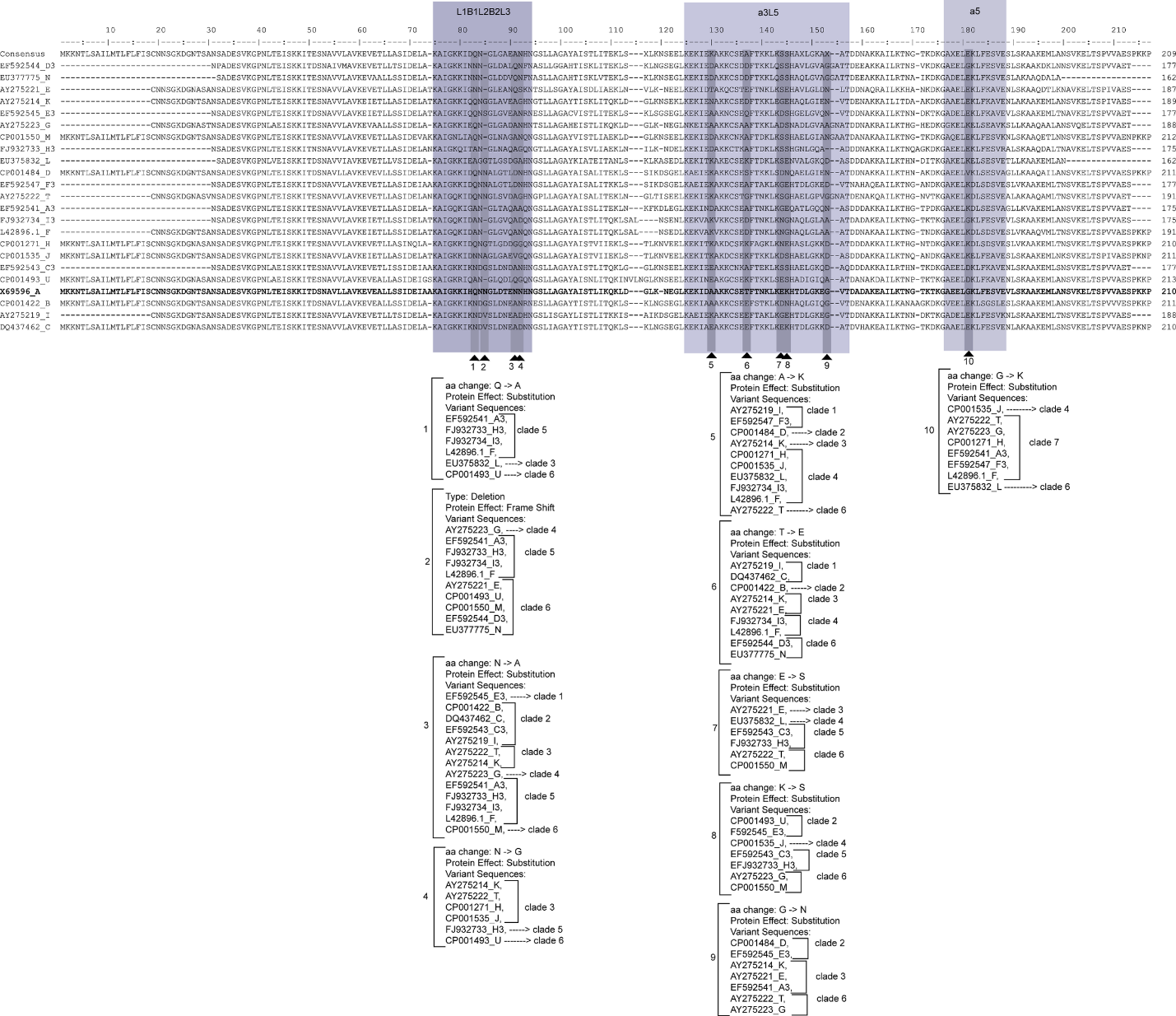


**Figure S1.** Pairwise amino acids sequences alignment of the 23 ospC alleles used by Baum and colleagues. The L1β1L2β2L3, α3L5 and α5 fragments are highlighted with a light gray color and the amino acids change sites with a dark gray color. The reference sequence (X69596_A) is highlighted in bold. The SNPs mutations are numbered from 1 to 10 and indicated by an arrowhead. The details of the aa change, protein effect (e.g., substitution, frame shift, truncation or extension) and the sequence variants are reported under each motif region. The ruler is displayed and the consensus sequence is provided. The corresponding phylogenetic clades are added for each sequence variant.
